# Immunotherapy of food allergy: what is effective?

**DOI:** 10.1186/2045-7022-1-S1-S67

**Published:** 2011-08-12

**Authors:** Hugh Sampson

**Affiliations:** 1 Mount Sinai School of Medicine, Pediatrics, New York, USA

## 

Food-induced anaphylaxis is the single leading cause of anaphylaxis seen in emergency departments. Consequently, a number of therapeutic strategies to treat food allergies are being pursued. Two non-allergen-specific therapies have been investigated in man: monoclonal anti-human IgE antibodies and a Chinese herbal formulation, FAHF-2. A trial with HU-901 anti-IgE was shown to raise the threshold of reactivity significantly to peanut in a study of peanut-allergic patients. FAHF-2, which was found highly effective in blocking anaphylaxis in a murine model of peanut allergy, was found safe in a phase I safety trial and is now being evaluated in a phase II efficacy trial. Other non-specific approaches showing promise in preclinical studies include the administration of Trichuris suis, Lactococcus lactis transfected with IL-10 or with IL-12 and food β-lactoglobulin, and a Toll-like receptor 9 agonist. A number of allergen-specific therapies are also being investigated in clinical trials: oral immunotherapy (OIT), sublingual immunotherapy (SLIT), epicutaneous immunotherapy (EPIT), modified recombinant peanut proteins within heat-killed E coli (EMP-123), and administration of baked (heat-denatured) foods. OIT appears to effectively "desensitize" the majority of patients treated, although adverse reactions remain problematic and whether "tolerance" can be induced remains to be determined. SLIT appears promising in initial trials with fewer adverse reactions, but whether tolerance can be established is not clear. EPIT and EMP-123 are in early clinical trials, although a small uncontrolled trial of milk-EPIT suggested a beneficial effect with minimal adverse symptoms. In one clinical trial, administering baked milk-containing products to children who tolerated this form of the food (~80% of milk-allergic children) was found to lead to "tolerance" in ~60% of the children over a 2 - 3 year period. Other approaches that have shown promise in preclinical studies include peptide (T-cell epitope) immunotherapy, plasmid immunotherapy, immunostimulatory sequence (CpG) conjugated proteins, Human immunoglobulin Fc-Fc fusion proteins, and mannoside-conjugated protein (BSA).

